# Application and Effectiveness of Telehealth to Support Severe Mental Illness Management: Systematic Review

**DOI:** 10.2196/mental.8816

**Published:** 2018-11-21

**Authors:** Sadie Lawes-Wickwar, Hayley McBain, Kathleen Mulligan

**Affiliations:** 1 Centre for Health Services Research School of Health Sciences City, University of London London United Kingdom; 2 East London National Health Service Foundation Trust London United Kingdom

**Keywords:** severe mental disorders, telehealth, technology, systematic review, mobile phone

## Abstract

**Background:**

People with severe mental illness (SMI) must receive early interventions to prevent mental health deterioration or relapse. Telecommunications and other technologies are increasingly being used to assist in health care delivery using “telehealth,” which includes telephones and mobile phones, computers, remote sensors, the internet, and other devices, to provide immediate real-time information to service users to improve the management of chronic health conditions. Some initial findings have suggested that technology could improve the quality of life of people with SMI.

**Objective:**

In this systematic review, we aimed to identify the various uses and efficacy of telehealth technology for SMI.

**Methods:**

We systematically searched electronic databases from inception to March 2016 (MEDLINE, EMBASE, PsycINFO, Cochrane Central Register of Controlled Trials, Allied and Complementary Medicine Database, Health Technology Assessment, CINAHL Plus, and NHS Economic Evaluations Database) to identify randomized controlled trials evaluating telehealth for adults with SMI published in English. Additional literature was identified through searching reference lists of key articles. The articles meeting the inclusion criteria were systematically reviewed and assessed for quality and risk of bias.

**Results:**

Our search identified 31 articles describing 29 trials as eligible for the review. The included studies evaluated the use of computers to deliver cognitive rehabilitation (15 trials), patient education (3 trials), and Web-based self-management interventions (2 trials) and to support consultations (1 trial). Virtual reality was used to simulate work and social situations (2 trials) and to deliver cognitive training (1 trial). Telephones were used to prompt service users to take medications (3 trials) and to report symptoms to their health care team (1 trial). Remote sensors were used to monitor medication use (1 trial). Telephone support was found effective in improving medication adherence and reducing the severity of symptoms and inpatient days. Computer-assisted cognitive rehabilitation was effective in improving cognitive function. The impact of telehealth on other outcomes was inconsistent. The results of this review should be taken in the context of varied quality in study design, with only 5 studies demonstrating a low risk of bias.

**Conclusions:**

A growing variety of telehealth technologies are being used to support the management of SMI. Specific technology types have been found to be effective for some outcomes (eg, telephone and remote medication monitoring for adherence to treatment), while other types of telehealth technologies (eg, delivery of patient education using computers) had no benefit over traditional nurse-based methods and were less acceptable to patients. Further research is warranted to establish the full potential benefits of telehealth for improving the quality of life in people with SMI, acceptability from the service user perspective, and cost-effectiveness. The findings of this review are limited by the poor quality of many of the studies reviewed.

## Introduction

Telecommunications and other technologies are increasingly being used to assist in health care delivery and are collectively known as “telehealth.” Telehealth is broadly defined as the use of applications in health care including telephones, mobile phones, computers, the internet, and audio and video processing to provide service users with immediate real-time information aimed at enhancing the management of their condition or its symptoms [1-3]. Telehealth has been found to be effective in managing a range of long-term conditions, including respiratory and cardiac diseases and diabetes [4-7]. Benefits include reductions in health service use [4-7], including hospitalization and emergency department visits, and improved clinical outcomes [4,7], for example, glycemic control in people with diabetes [4]. Some initial evidence also suggests that technology is found to be acceptable by users to support health management, particularly in terms of convenience [8]. While studies measuring the acceptability of using technology to support health care are still emerging, a review by Or and Karsh [9] identified specific factors that predict the acceptability of telehealth for long-term conditions, including younger age, higher levels of education, prior experience, perceived usefulness and ease of use, and satisfaction [9].

Severe mental illness (SMI) is commonly defined by the presence of persistent and extensive functional disability [10] and includes psychotic disorder, schizophrenia, schizoaffective disorder, major depressive disorder, and bipolar disorder. Previous systematic reviews have evaluated either the use of one specific type of telehealth, for example, telephone prompts, to promote appointment attendance [11,12] or the use of telehealth more broadly in a specific mental illness. Some initial findings have suggested that technology-based prompts could improve quality of life and SMI symptoms in people with SMI. However, the quality of the evidence has been found to be low [12]. Furthermore, the review of telephone prompts was published in 2009, and given the rise in the use of technology, an updated review is due. In 2 reviews, a range of applications for general mental health, including dementia, child psychiatry, suicide prevention, substance misuse, and psychotic disorders, were evaluated [13,14] and evidence for benefits to mood, trauma-related symptoms, and suicide attempts and better medication adherence was found. However, a review of the range of available telehealth technologies and their use to support people with SMI does not exist. Given the current multitude of available telehealth technologies and the rapid increase in their use, a further review is required to identify the range of uses for telehealth in the context of SMI and whether they can lead to increased service user engagement and improved psychological and clinical outcomes across SMIs.

The aims of this review are to (1) identify and describe how telehealth interventions for people with SMI have been implemented to date and (2) synthesize the evidence in relation to the effectiveness or efficacy of available interventions.

## Methods

### Study Eligibility

Studies were selected for inclusion in the review if they met the following criteria:

1. Adults aged ≥18 years diagnosed with SMI defined as psychotic disorder, schizophrenia, schizoaffective disorder, major depressive disorder, and bipolar disorder as defined by Johnson [10]. If studies included service users with and without SMI, only data that could be extracted for users with SMI were included. Studies for which it was unclear whether participants in a population of “young people” were aged ≥18 years were not included.
2. Randomized controlled trials (RCTs) available in English language.
3. Interventions that used telehealth technology targeted to improve the management of SMI.
4. Articles measuring the following outcome measures were considered for this review:
  - General or disease-specific psychological or psychosocial outcomes, including quality of life or mood, using generic or disease-specific validated tools.
  - Clinical outcomes including reduction in psychotic symptoms, introduction of new antipsychotics, increased intensity of medication, hospital readmission, mortality rates, and progression of SMI.
  - Attendance as an outpatient or in primary care.
  - Adherence to treatment, including medication or recommended psychological support.

Studies were excluded if SMI was caused by dementia or brain injury, they were not available in English, they investigated telecare or social care technology such as remote sensors for falls, the intervention focused on carers or health care professionals rather than service users, participants were not randomized, technology was not the primary focus of the intervention, and participants had major depressive disorder or other mood disorders without psychosis.

### Search Strategy

Electronic searches using the following databases were conducted from inception to March 2016: MEDLINE, EMBASE, PsycINFO, Cochrane Central Register of Controlled Trials, Allied and Complementary Medicine Database, Health Technology Assessment, CINAHL Plus with Full Text via EBSCOhost, and NHS Economic Evaluations Database by searching “all fields” using the following search terms: TELEHEALTH, TELE*, TECHNOLOGY, E?HEALTH, M?HEALTH, ONLINE, WEB*, INTERNET, COMPUTER, MOBILE, APP, VIRTUAL CONSULTATION, PHONE, POCKET PC, IPHONE, SHORT MESSAGE SERVICE, SMS, TEXT MESSAG*, WIRELESS, SMARTPHONE, REAL-TIME, ELECTRONIC DIAR* *and* INTERVENTION, PROGRAM*, THERAPY, SUPPORT, EDUCATION, TRAINING *and* SEVERE MENTAL ILLNESS, MENTAL DISORDER, PSYCHOTIC DISORDER, MOOD, AFFECTIVE DISORDER, PERSONALITY DISORDER, BIPOLAR DISORDER, SCHIZOPHRENIA, DEPRESSION, SCHIZOAFFECTIVE DISORDER *and* RANDOMI?ED, TRIAL, CLINICAL TRIAL, COMPARATIVE STUDY, SYSTEMATIC REVIEW, META-ANALYSIS, REVIEW, CROSSOVER PROCEDURE, DOUBLE BLIND*, and SINGLE BLIND*.

The first author checked titles, and all 3 authors reviewed abstracts to exclude any irrelevant articles. Full texts of the remaining articles were obtained; all 3 authors screened these. Any disagreements were discussed within the research team to reach consensus. References were searched for additional papers.

### Data Extraction and Management

The first author extracted data using a standardized form developed by the Cochrane Collaboration [15], which included information on the following: study characteristics (including aim and design), participants (including population description, inclusion and exclusion criteria, and baseline imbalances), features of the intervention and comparison groups (including description, timing, and providers), outcome measures, statistical methods used in analysis, results, and conclusions.

### Risk of Bias

The Cochrane Collaboration tool to assess the risk of bias in RCTs [16] was used. The Cochrane risk of bias tool includes a list of potential sources of bias in clinical trials in 7 main areas: random sequence generation, allocation concealment, blinding of participants and personnel, blinding of outcome assessment, incomplete outcome data, selective reporting, and other bias [16]. The rating scale for each area of bias ranges from “low risk of bias” to “high risk of bias” and an option of “unclear risk of bias” for studies that do not provide enough details to be able to make a clear judgment [16]. Two of the review authors (SLW, KM) independently judged each article for risk of bias, discussing disagreements until a consensus was reached.

## Results

### Studies identified

**Figure 1:**
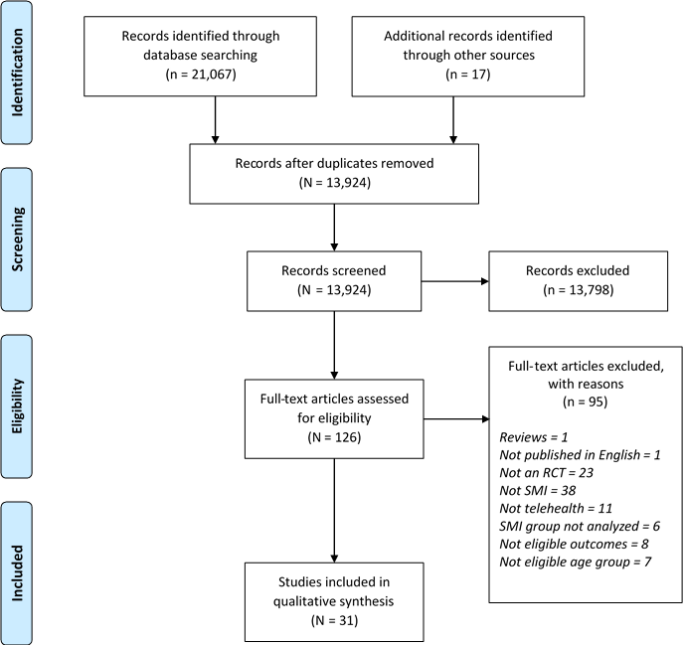
Preferred Reporting Items for Systematic Reviews and Meta-Analyses flow diagram of each stage of the study screening and selection process. SMI: severe mental illness, RCT: randomized controlled trial.

Electronic searches identified 13,907 unique articles, and reference searches of key articles identified a further 17 potentially eligible articles. The inclusion criteria were met by 31 articles, which have been presented in a Preferred Reporting Items for Systematic Reviews and Meta-Analyses [17] diagram (Figure 1). A meta-analysis was not performed due to the heterogeneity of the interventions, including their methods of delivery and the outcome measures of the included studies. The characteristics of the final 31 articles can be seen in Multimedia Appendix 1.

The 31 articles reported 29 trials. A total of 17 trials focused on people with schizophrenia, 9 on people with schizophrenia or schizoaffective disorder or psychotic disorder, and 3 on people with bipolar disorder. A range of telehealth devices were used with varying aims. Computers were used to improve cognitive functioning and disease-specific knowledge; websites aimed to improve psychosocial functioning; hand-held devices were used to improve communication with medical staff; telephones were used to improve medication adherence and disease-specific symptoms; virtual reality (VR) aimed to improve social and work-related functioning; and electronic medication dispensers aimed to improve adherence. The specific outcome measures included medication adherence (4 studies), social functioning (including work behavior; 5 studies), health care utilization (4 studies), neurocognitive functioning (16 studies), knowledge about medication or SMI (2 studies), self-esteem (3 studies), self-efficacy (1 study), quality of life (4 studies), mood (4 studies), insight into condition (1 study), perceptions of deprivation of liberty (1 study), satisfaction with life or treatment (5 studies), and illness perceptions (1 study). The 29 included trials recruited a total of 4338 participants. Sample sizes ranged from 29 to 507. Details about each intervention can be seen in Multimedia Appendix 2.

### Risk of Bias

Results for the risk of bias assessment can be found in Multimedia Appendix 3. Of note, only 5 studies were rated as high quality. One study demonstrated a particularly high and, at times, unclear risk of bias for the majority of sources of bias [18]. Several studies did not provide enough details to be able to make a clear judgment that they had not introduced bias into their findings.

### Intervention Effectiveness

Results for each of the 31 studies can be found in Multimedia Appendix 4. A description of the findings is presented in the following sections.

#### Cognitive Outcomes

Of the 31 articles reviewed, 20 reported the impact of telehealth for SMI on cognitive outcomes. Neurocognitive functioning was measured in 18 studies and encompassed memory, attention, executive functioning, and visual perception. Of these, 15 studies measured the impact of computer-assisted cognitive rehabilitation (CACR) on neurocognitive functioning [19-33], of which 11 found statistically significant improvements [21-25,27,29-32,34]; furthermore, 2 of the 18 studies evaluated the use of VR for cognitive rehabilitation [34] and vocational rehabilitation [35] for adults with schizophrenia. VR was found to have a significant effect on cognitive functioning in both studies [34,35]. One study [36] evaluated the use of offline personalized computer-based health education compared with nurse-delivered health education and a combination of both interventions, but it found no significant differences from baseline to follow-up in any of the trial arms on measures of neurocognitive functioning or knowledge about schizophrenia [36,37].

To summarize the findings for cognitive outcomes, 11 of 15 studies found CACR to be beneficial for cognitive outcomes, and VR-based cognitive and vocational training was found to be effective for cognitive function, while computer-based patient education was found to have no benefit for cognitive outcomes over nurse-delivered education.

#### Psychosocial Outcomes

Of the 31 articles reviewed, 21 reported the impact of telehealth on psychosocial outcomes. These included positive and negative symptoms [22,23,25,28,29,31,37-39], social adaptation including social adjustment and cognition [22,25,28,29,32,40-42], quality of life [21,22,37,39-41,43], mood [32,37,40,44,45], satisfaction with care [43], self-esteem [19,25,40], self-efficacy [35], illness perceptions [40], insight [36], and perceptions of deprivation of liberty [46].

##### Positive and Negative Symptoms

The effectiveness of CACR on schizophrenia symptoms was evaluated in 6 studies [19,22,23,25,28,31], of which 4 did not find any effect; however, 2 found a beneficial effect on negative symptoms [19,28]. Positive and global symptoms were also measured in 1 study, finding a beneficial effect on positive symptoms but not global symptoms [28].

Telephone-based nurse support reduced positive schizophrenia symptoms compared with a control group, but no differences were observed for negative or global schizophrenia symptoms [37]. Telemonitoring [38] had a beneficial effect on positive and negative schizophrenia symptoms over standard care, although a nontechnology “pill counting” group also showed improved symptoms. Computer-mediated structured consultations [39] did not have any impact on symptoms.

##### Social Adaptation

The included trials measured several different aspects of social adaptation. These included social cognition, social adjustment, and social professional and family functioning. Of the 31 studies, 8 evaluated the impact of telehealth on social adaptation, encompassing social adjustment and social cognition, of which 3 reported a benefit. Of 5 trials of CACR, 2 reported a beneficial effect on the social and living conditions subscales of the Health of the Nation Outcome Scales [28,47], social adjustment (defined as role performance), and social cognition (defined as awareness of relationships), which were measured using a combination of existing outcome measures [29]. However, others found no effect of CACR on social autonomy [22], social professional and family functioning [32], or social cognition as measured using the MATRICS Consensus Cognitive Battery [25,48].

A Web-based self-management intervention [41] improved social functioning, but a Web-based cognitive behavioral therapy (CBT)-based intervention (alone or with peer support) [40] did not affect the perceptions of stigmatization. Using VR to deliver social skills training [42] did not differ from social skills training with role play on social problem solving, social adjustment, or stigma.

##### Quality of Life

The impact of telehealth for SMI on quality of life was reported in 7 articles. Of these, 3 studies found a significant benefit of telehealth for SMI. CACR had a significant effect on the overall quality of life and a self-directedness subscale in 1 study [21] but not in another [22]. The use of computer-mediated structured consultations (“DIALOG”) improved the quality of life at a 12-month follow-up [39]. Service users with a shorter duration of illness and a better “perceived helping alliance” with key workers prior to receiving DIALOG improved more in subjective quality of life than with standard care [43]. Hansson et al [43] also found that people with more severe negative schizophrenia symptoms prior to the intervention experienced a greater improvement in the quality of life after receiving DIALOG. Web-based self-management [41] had a significant effect on both physical and psychological quality of life, but nurse-based telephone support [37] was not effective.

Satisfaction with life was measured in 2 studies. There were no significant differences between “MoodSwings,” “MoodSwings” plus Web-based peer support, and a standard care control group on satisfaction with life [40]. Similarly, there was no difference between an information technology education group and a standard care group on satisfaction with life [46].

##### Mood

A nurse-delivered monthly telephone monitoring intervention [44,45] reduced depressive symptoms for bipolar disorder after 12 months, but this effect was not maintained at the 2-year follow-up. Furthermore, a significant reduction in mania was found at the 2-year follow-up, but not at the initial 12-month assessment. No benefit was found for CACR [32], a Web-based CBT–based intervention [40], or a telephone-based nurse support intervention [37] over a no treatment control. The control condition in Proudfoot et al’s [40] study, which involved information about bipolar disorder being emailed to participants weekly, is noteworthy.

##### Satisfaction With Health Care

The effectiveness of telehealth on satisfaction with health care was measured in 1 trial. Computer-mediated consultations improved treatment satisfaction [43].

##### Self-Esteem

The impact of telehealth for SMI on self-esteem was measured in 3 studies. Self-esteem was not improved by CACR [19,25], or by a Web-based CBT–based intervention [40].

##### Self-Efficacy

The 1 study that measured self-efficacy found no differences between VR vocational training, therapist-administered vocational training, and a nontraining control group in terms of self-efficacy for performing work-related tasks [35]. None of the computer-based education trials found a significant impact on insight into schizophrenia [36] or perceived deprivation of liberty among adults with schizophrenia [46].

To summarize the findings for psychosocial outcomes, CACR was found to have no effect on schizophrenia symptoms in the majority of studies that measured this outcome and had no effect on mood over standard care. Of the 3 studies that measured the impact of CACR on psychosocial adjustment, 2 found an improvement. Telephone support was found to reduce the severity of positive symptoms and depressive symptoms in schizophrenia, and remote telemonitoring was found to improve global schizophrenia symptoms over pill counting alone. Computer-mediated structured consultations and Web-based self-management for bipolar disorder were both found to lead to improvements in quality of life. While Web-based patient education improved social functioning, it had no effect on mood. VR had no benefits in improving nonverbal social skills over traditional face-to-face training.

#### Behavioral Outcomes

Of the 31 studies, 10 measured the effectiveness of telehealth interventions on behavioral outcomes. Outcomes included adherence to treatment [18,37,38,49]; health care utilization, including number of inpatient days and amount of antipsychotic medication taken [38,41,44,50]; work habits [23,35]; and conversational skills, including nonverbal skills [42].

##### Adherence to Treatment

Of the 4 studies that measured adherence to treatment, significant improvements were found with nurse-based telephone support [37,49] and telemonitoring [38], but not for computer-based medication education [18].

##### Health Care Utilization

Of the 4 studies that evaluated the use of telehealth in health care utilization, all found a significant positive effect. Remote medication monitoring [38] led to fewer medical and emergency visits than pill counting alone and standard care. Telemonitoring of symptoms [50] led to significantly fewer inpatient days compared with a control group that received standard care. Simon et al [44] found better adherence to atypical antipsychotic use and increased attendance for medication management visits in service users who received nurse-based telephone support. Todd et al [41] reported that medication and service use reduced, on average, in the self-management website group and increased, on average, for the standard group, although statistical analysis of the difference was not reported.

##### Work Habits

Of the 2 studies that measured work habits, which involved direct observation of work behavior and an interview with the work supervisor, both found the telehealth intervention to be effective. CACR [23] had a significant effect on work habits, and VR [35] was as effective as therapist-delivered vocational training for work-related tasks, with a significant improvement seen in both groups.

##### Conversational Skills

Park et al [42] found that enabling service users to practice their social skills using VR after receiving social skills training had a significant effect on their conversational skills and assertiveness compared with a face-to-face role-playing group (without VR), although the role-playing group showed greater improvement in nonverbal skills.

To summarize the findings for behavioral outcomes, telephone support and remote telemonitoring consistently led to improvements in medication adherence and reduced inpatient days, while computer-based education had no effect. Telemonitoring and Web-based CBT–based self-management both led to reduced emergency health care visits. CACR and VR were both effective for improving work behavior and VR improved assertiveness in social situations over traditional face-to-face training.

#### Acceptability

Of note, only 1 of the included studies formally measured whether participants found the intervention they received to be acceptable. Frangou et al [38] measured acceptability, ease of use within routine care, and perceived effectiveness qualitatively from the perspective of service users and their health care professionals. There was unanimous agreement among service users that the telemonitoring intervention helped them manage their condition and was easy to use and incorporate into daily life, and caregivers shared these views [38]. While Park et al [42] did not report measuring “acceptability,” they did record “interest in participation” using a 2-item questionnaire, which evaluated participants’ interest in the session they received and their expectations for the next session. The VR group scored higher on interest in participation than the traditional social skills training group [42], suggesting a preference for technology. Jones et al [36] similarly did not refer to “acceptability” but assessed participants’ opinions about the computer-based education intervention combined with nurse-based education versus computer-based or nurse-based education alone. Significantly more service users in the nurse-based education group perceived the information they received as definitely relevant to them compared with the computer-delivered education group and the combination group, despite the nurse-delivered intervention providing the same content as the computer system [36].

One indicator of acceptability may be significant dropout rates from the intervention group. Most of the included studies did not present a substantial dropout rate, although not all studies adequately reported the reasons for dropout. Of the studies reporting a substantial dropout rate, Proudfoot et al [40] reported a higher dropout from the website condition (30%) than from the website plus peer support condition (19%). Jones et al [36] reported substantial dropouts from the computer-delivered (41%) and nurse-delivered education (46%) conditions compared with the combined condition (29%). Reasons for dropout from the computer education group included refusal to continue taking part, the intervention being unsafe, and physical problems limiting the ability to continue with the intervention [36]. Rass et al [26] also reported a higher dropout from a CACR group (19%) than from either control condition (0%), including 3 participants who stopped attending the intervention and 2 who did not complete final follow-up assessments. In a longitudinal study assessing telephone monitoring plus a structured group psychoeducational program, while the authors reported a high level of contact in the telephone element of the intervention (85% of participants completed 12 or more telephone contacts), group participation dropped substantially to 51% after 12 months [45], suggesting that a group-based education intervention may be less acceptable than telephone monitoring in adults with bipolar disorder.

## Discussion

### Principal Results

The aims of this review were (1) to identify which, and how, telehealth interventions have been trialed for people with SMI and (2) to synthesize the evidence in relation to the effectiveness or efficacy of these interventions. This review identified 31 articles describing 29 trials, including a total of 4338 participants with schizophrenia, schizoaffective disorder, psychotic disorder, and bipolar disorder. The studies in the included articles evaluated the use of computers for cognitive rehabilitation, patient education, consultations with key workers, and interactive Web-based CBT–based self-management interventions; the use of VR to simulate work and social situations and to deliver cognitive training; the use of telephones to prompt medication use and to report SMI symptoms to health care teams; and the use of remote sensors to monitor medication use.

This review found evidence for using some types of technology to support the management of SMI while finding that not all technology is effective, depending on the outcome of interest. Interventions containing telephone support from the medical team, including phone calls and short message service text messaging prompts about medication, were consistently found to be effective in improving medication adherence while also reducing the severity of mania symptoms and reducing inpatient days. CACR was found to be effective in improving cognitive outcomes in schizophrenia in most, but not all, studies. A Web-based CBT-based self-management intervention for bipolar disorder was found to be effective at improving quality of life and social functioning. The use of VR was found to be effective in improving work-related behavior, conversational skills, assertiveness, and cognitive functioning, although face-to-face social skills training was found to be more effective in improving nonverbal social skills than VR-delivered training. Computers appeared not to have a superior benefit for delivering patient education over traditional nurse-delivered education, and in fact, participants preferred the nurse-delivered method. This finding is perhaps reflective of the nature of interactions with health care staff, which can provide a personal and individualized approach to supporting people with SMI, for example, by offering opportunities like discussing diagnoses and modeling nonverbal social cues. Hand-held devices to support health care consultations were found to be effective in improving quality of life and satisfaction with care, but not in improving positive or negative schizophrenia symptoms. Telemonitoring was found to be effective in improving medication adherence and also led to fewer medical and emergency visits, including inpatient days, while simultaneously improving schizophrenia symptoms.

Notably, 5 studies demonstrated a low risk of bias for 6 out of the 7 sources of bias listed in the Cochrane risk of bias tool [28,39,41,43,44], and 6 studies demonstrated a low risk of bias for 5 out of the 7 sources [23,37,40,45,49,50]. One study demonstrated a particularly high and, at times, unclear risk of bias for the majority of sources of bias [18]. Several studies did not provide enough details to be able to make a clear judgment that they had not introduced bias into their findings, particularly in reference to whether participants were assigned to groups using an adequate randomization method and whether allocation had been truly concealed. The findings of this review should, therefore, be viewed in light of the potential bias introduced into the findings of some of the studies included.

The variety of comparison groups employed in the studies included in this review is also noteworthy. In the majority of articles (n=17), the authors reported that the comparison group received standard care or treatment as usual. This may have differed across trials. For example, in the context of inpatient care, this included medication and attendance at routine therapy groups [33], but in the context of community care, this might have included medication, physician visits, and support from available community centers [49]. However, for 9 of the studies, the control group received a comparative intervention, consisting of health care professional-delivered education about their condition [18,29], text-delivered education [40], therapist-delivered skills training or psychosocial interventions [28,35,42], or computer-delivered activity, such as computer games or education about their condition [21,25,36].

### Comparison With Prior Work

This review supports the findings of previous reviews evaluating cognitive training in schizophrenia [51,52], although evidence suggests that cognitive training is effective regardless of whether it is delivered via computer technologies or noncomputerized psychological interventions [51]. Substantial variations in the intensity and duration of the interventions that used software to support neurocognitive training may have impacted the results of these studies. If training was particularly intensive, for example, half a day or delivered over several months, it is possible that this might have overburdened participants with SMI, leading to a lack of significant findings, or reduced participants’ attention abilities. A recent meta-analysis suggests that as little as 5-15 hours of cognitive remediation could be sufficient for improving cognitive outcomes in people with schizophrenia [52], suggesting that more intensive cognitive training may not be necessary and that future interventions should be designed with this in mind.

CACR was most commonly assessed offline using personal computers and CD-ROM software, with little change in the software used over time. With the rise in more sophisticated technologies, including VR, which in this review was found to improve social and neurocognitive outcomes in people with SMI, perhaps we can expect to see VR interventions delivered more widely to support SMI outcomes in the coming years. VR has the potential to provide usable and safe, ecologically valid assistance in the management of SMI, as already found in general health care [53]. In particular, our findings support a recent systematic review of VR for people with SMI, which was found to be more interesting training than control conditions [54].

We identified surprisingly few studies that had evaluated Web-based interventions to support individuals with SMI. Educational websites were not found to be effective for improving knowledge about medication, adherence, self-esteem, insights into SMI, or deprivation of liberty over traditional nurse-delivered education. However, when websites provided more interactive elements than traditional education, such as peer support or CBT-based self-management techniques, improvements in quality of life, mood, and social adaptation were seen. Further research on the psychosocial benefits of Web-based interventions for people with SMI is suggested, particularly given the small number of studies to date and the growing evidence for such interventions in common mental health disorders, including anxiety and depression [55,56]. Web-based interventions will, however, need to take the specific needs of people with SMI into account to improve accessibility. For example, service users often report that common website design guidelines produce websites that are confusing for them to use, particularly in the presence of cognitive deficits [57].

The finding that telephones were effective for promoting adherence to medication use and attendance at health care appointments was consistent with a recent systematic review [58] of mobile phone-based technologies for supporting general health care and another review for the use of remote technology in SMI [58]. Further evidence suggests that the use of telephones has a beneficial impact on adherence over interventions that do not use technology, for example, psychoeducation for patients who are nonadherent due to forgetfulness [59]. While telephone-based interventions may not consistently improve clinical factors associated with SMI, the findings of the studies included in this review give early promise to the use of telephones in supporting adults with SMI to manage their medication. With advances in the function of telephones, including smartphone apps, this provides further opportunities for supporting people with SMI in the future. One might have expected more interventions, however, as Bakker et al [60] recently emphasized, mental health apps have not to date utilized the designs made available by physical health and social networking apps nor have the hundreds of apps available been tested using formal experimental methods.

The present review also found that the use of telephones improved patients’ attitudes toward using their treatment as well as their quality of life. Leach and Christensen [61] suggest that telephones are acceptable and cost-effective uses of technology to support health care due to their accessibility and convenience. A large-scale survey of users of mental health services in the United States in 2013 found that 72% users reported owning a mobile device, and both users and nonusers expressed an interest in future services being offered through mobile devices [62], suggesting that accessing a mobile device may not pose a barrier to people with SMI.

The positive findings regarding the use of telemonitoring to improve global schizophrenia symptoms, medication adherence, and medical and emergency visits are consistent with the reviews that have found positive effects of telemonitoring for managing chronic health conditions, including heart failure [63] and respiratory conditions [64]. Furthermore, a recent review of the use of remote technology for SMI found this to be a feasible and acceptable method of health care delivery [65]. These are positive initial results for a form of telehealth that was found to be acceptable from both the service user and health care perspective, and future research evaluating the use of telemonitoring for SMI could have implications for the delivery of future services for people with SMI.

The finding that computer-mediated consultations led to improvements in quality of life and reduced unmet need in service users with SMI is promising for a novel use of technology, where there is currently a limited evidence base. One recent qualitative study reports that health care professionals perceive tablet computers to fill a need between smartphones and desktop computers and have some value in supporting consultations with patients [66]. Tablet computers offered support in structuring consultations, ensuring that patients’ priorities were discussed [39]; thus, patients might feel their needs have been better dealt with.

Few studies evaluated the use of remote medication telemonitoring, computer-mediated consultations, or Web-based self-management resources, suggesting these are novel technologies for managing SMI, although all were found to be effective for improving psychosocial and behavioral outcomes. We were surprised to find the limited use of these more advanced technologies in an SMI context given the rise in their use to support broader health care in recent years, for example, Web-based self-management programs for diabetes [67]. We suggest that future research should seek to establish the full potential benefits of these novel uses of telehealth for improving the management of SMI.

Of note is the varied quality of the studies included in the review, with only 5 studies rated as high quality. This suggests that high levels of bias were potentially introduced into the results of these studies. Of particular note is the small sample size of the majority of articles in this review, with 19 of the total 31 studies having samples of <100 participants. Furthermore, the type of comparison or control group employed might have had an influence on the effect size of the intervention evaluated, and the variety of comparison groups included in this review is noteworthy. The generalizability of the findings from many of these studies is, therefore, limited.

### Limitations

To our knowledge, this is the first systematic review of RCTs across a range of telehealth technology interventions delivered to people with SMI. The studies included in this review measured a plethora of outcomes using heterogeneous measures and a range of interventions, making meta-analysis of the results impossible. In addition, we excluded articles that had not been published in English. It is possible, therefore, that we excluded relevant foreign language articles from this review. One author performed the data extraction process; while this was not verified by a second author, all authors were involved in the screening process, and 2 authors independently completed the risk of bias assessment. Thus, several stages of the review process were validated by more than 1 author.

The lack of studies that formally evaluated the acceptability of telehealth interventions is noteworthy. Of those that did measure the opinions of their participants, telehealth was found to be acceptable, particularly in comparison with more traditional face-to-face methods of the delivery. However, this may depend on whether a face-to-face element to the intervention is also offered, as it has been suggested that this may be preferable over unguided interventions [68]. If telehealth is to be developed to support the care of people with SMI, it is important that acceptability of the interventions is considered as part of the evaluation and formally measured. From a health-commissioning perspective, it is also possible that telehealth delivery costs are higher than those of usual care [1]. Cost-effectiveness was not formally evaluated by the studies included in this review; thus, it is unclear whether this is also the case with interventions in SMI. Future studies should evaluate the costs of using technology to support the management of SMI.

### Conclusions

This systematic review has identified a range of ways in which telehealth has been used to support the management of SMI and its symptoms. The studies found some strengths of cognitive remediation for schizophrenia, whether delivered through a personal computer and CD-ROM or VR. The use of telephone support from the medical team was consistently found to be effective for improving medication adherence and reducing the severity of symptoms and inpatient days. Few studies evaluated the use of remote medication telemonitoring, VR, Web-based self-management, and hand-held devices, suggesting that these are novel technologies for managing SMI, although all were found to be effective in improving some psychosocial and behavioral outcomes. Patient preferences should be assessed and accommodated, as some may prefer traditional methods of delivery with health care staff over computer-based methods. Given the poor quality of all but 5 of the included trials and that few studies have evaluated the acceptability and cost-effectiveness of using technology to support people with SMI, further studies are needed to establish the potential benefits to these areas.

## Appendix

Multimedia Appendix 1 Characteristics of participants included in the eligible trials.

Multimedia Appendix 2 Descriptions of the interventions included in the review.

Multimedia Appendix 3 Risk of bias assessment.

Multimedia Appendix 4 Results of included studies.

## Abbreviations

CACR: computer-assisted cognitive remediation
CBT: cognitive behavioral therapy
RCT: randomized controlled trial
SMI: severe mental illness
VR: virtual reality

## References

[1] Henderson C, Knapp M, Fernández José-Luis, Beecham J, Hirani SP, Cartwright M, Rixon L, Beynon M, Rogers A, Bower P, Doll H, Fitzpatrick Ray, Steventon Adam, Bardsley Martin, Hendy Jane, Newman Stanton P, Whole System Demonstrator evaluation team (2013). Cost effectiveness of telehealth for patients with long term conditions (Whole Systems Demonstrator telehealth questionnaire study): nested economic evaluation in a pragmatic, cluster randomised controlled trial. BMJ.

[2] Nickelson DW (1998). Telehealth and the evolving health care system: Strategic opportunities for professional psychology. Professional Psychology: Research and Practice.

[3] VandenBos GR, Williams S (2000). The Internet versus the telephone: What is telehealth anyway?. Professional Psychology: Research and Practice.

[4] Polisena J, Tran K, Cimon K, Hutton B, McGill S, Palmer K (2009). Home telehealth for diabetes management: a systematic review and meta-analysis. Diabetes Obes Metab.

[5] Polisena J, Tran K, Cimon K, Hutton B, McGill S, Palmer K, Scott RE (2010). Home telehealth for chronic obstructive pulmonary disease: a systematic review and meta-analysis. J Telemed Telecare.

[6] Paré Guy, Jaana M, Sicotte C (2007). Systematic review of home telemonitoring for chronic diseases: the evidence base. J Am Med Inform Assoc.

[7] Barlow J, Singh D, Bayer S, Curry R (2007). A systematic review of the benefits of home telecare for frail elderly people and those with long-term conditions. J Telemed Telecare.

[8] Hommel KA, Hente E, Herzer M, Ingerski LM, Denson LA (2013). Telehealth behavioral treatment for medication nonadherence: a pilot and feasibility study. Eur J Gastroenterol Hepatol.

[9] Or CK, Karsh BT (2009). A systematic review of patient acceptance of consumer health information technology. J Am Med Inform Assoc.

[10] Johnson DL (1997). Overview of severe mental illness. Clin Psychol Rev.

[11] Reda S, Makhoul S (2001). Prompts to encourage appointment attendance for people with serious mental illness. Cochrane Database Syst Rev.

[12] Kauppi K, Välimäki Maritta, Hätönen Heli M, Kuosmanen LM, Warwick-Smith K, Adams CE (2014). Information and communication technology based prompting for treatment compliance for people with serious mental illness. Cochrane Database Syst Rev.

[13] Hailey D, Roine R, Ohinmaa A (2008). The effectiveness of telemental health applications: a review. Can J Psychiatry.

[14] Van Der Krieke L, Wunderink L, Emerencia AC, de Jonge P, Sytema S (2014). E-mental health self-management for psychotic disorders: state of the art and future perspectives. Psychiatric Services.

[15] The Cochrane Collaboration (2014). Data collection form for intervention reviews: RCTs only.

[16] Higgins JPT, Altman DG, Gøtzsche Peter C, Jüni Peter, Moher D, Oxman AD, Savovic Jelena, Schulz KF, Weeks L, Sterne JA, Cochrane Bias Methods Group, Cochrane Statistical Methods Group (2011). The Cochrane Collaboration's tool for assessing risk of bias in randomised trials. BMJ.

[17] Moher D, Liberati A, Tetzlaff J, Altman DG, PRISMA Group (2009). Preferred reporting items for systematic reviews and meta-analyses: the PRISMA statement. PLoS Med.

[18] Madoff SA, Pristach CA, Smith CM, Pristach EA (1996). Computerized medication instruction for psychiatric inpatients admitted for acute care. MD Comput.

[19] Bellucci DM, Glaberman K, Haslam N (2003). Computer-assisted cognitive rehabilitation reduces negative symptoms in the severely mentally ill. Schizophrenia research.

[20] Burda P, Starkey TW, Dominguez F, Vera V (1994). Computer-assisted cognitive rehabilitation of chronic psychiatric inpatients. Computers in Human Behavior.

[21] Cavallaro R, Anselmetti S, Poletti S, Bechi M, Ermoli E, Cocchi F, Stratta P, Vita A, Rossi A, Smeraldi E (2009). Computer-aided neurocognitive remediation as an enhancing strategy for schizophrenia rehabilitation. Psychiatry Res.

[22] d'Amato T, Bation R, Cochet A, Jalenques I, Galland F, Giraud-Baro E, Pacaud-Troncin M, Augier-Astolfi F, Llorca P, Saoud M, Brunelin J (2011). A randomized, controlled trial of computer-assisted cognitive remediation for schizophrenia. Schizophr Res.

[23] Lee WK (2013). Effectiveness of computerized cognitive rehabilitation training on symptomatological, neuropsychological and work function in patients with schizophrenia. Asia Pac Psychiatry.

[24] Mak M, Samochowiec J, Tybura P, Bieńkowski P, Karakiewicz B, Zaremba PL, Mroczek B (2013). The efficacy of cognitive rehabilitation with RehaCom programme in schizophrenia patients. The role of selected genetic polymorphisms in successful cognitive rehabilitation. Ann Agric Environ Med.

[25] Keefe RSE, Vinogradov S, Medalia A, Buckley PF, Caroff SN, D'Souza DC, Harvey PD, Graham KA, Hamer RM, Marder SM, Miller DD, Olson SJ, Patel JK, Velligan D, Walker TM, Haim AJ, Stroup TS (2012). Feasibility and pilot efficacy results from the multisite Cognitive Remediation in the Schizophrenia Trials Network (CRSTN) randomized controlled trial. J Clin Psychiatry.

[26] Rass O, Forsyth JK, Bolbecker AR, Hetrick WP, Breier A, Lysaker PH, O'Donnell BF (2012). Computer-assisted cognitive remediation for schizophrenia: a randomized single-blind pilot study. Schizophr Res.

[27] Sartory G, Zorn C, Groetzinger G, Windgassen K (2005). Computerized cognitive remediation improves verbal learning and processing speed in schizophrenia. Schizophr Res.

[28] Vita A, De Peri L, Barlati S, Cacciani P, Deste G, Poli R, Agrimi E, Cesana BM, Sacchetti E (2011). Effectiveness of different modalities of cognitive remediation on symptomatological, neuropsychological, and functional outcome domains in schizophrenia: a prospective study in a real-world setting. Schizophr Res.

[29] Hogarty GE, Flesher S, Ulrich R, Carter M, Greenwald D, Pogue-Geile M, Kechavan M, Cooley S, DiBarry AL, Garrett A, Parepally H, Zoretich Rebecca (2004). Cognitive enhancement therapy for schizophrenia: effects of a 2-year randomized trial on cognition and behavior. Arch Gen Psychiatry.

[30] Kurtz M, Seltzer JC, Shagan DS, Thime WR, Wexler BE (2007). Computer-assisted cognitive remediation in schizophrenia: what is the active ingredient?. Schizophr Res.

[31] Dickinson D, Tenhula W, Morris S, Brown C, Peer J, Spencer K, Li L, Gold JM, Bellack AS (2010). A randomized, controlled trial of computer-assisted cognitive remediation for schizophrenia. Am J Psychiatry.

[32] Hermanutz M, Gestrich J (1991). Computer-assisted attention training in schizophrenics. A comparative study. Eur Arch Psychiatry Clin Neurosci.

[33] Benedict RH, Harris AE, Markow T, McCormick JA, Nuechterlein KH, Asarnow RF (1994). Effects of attention training on information processing in schizophrenia. Schizophr Bull.

[34] Chan CL, Ngai EK, Leung PK, Wong S (2010). Effect of the adapted Virtual Reality cognitive training program among Chinese older adults with chronic schizophrenia: a pilot study. Int J Geriatr Psychiatry.

[35] Tsang MMY, Man DWK (2013). A virtual reality-based vocational training system (VRVTS) for people with schizophrenia in vocational rehabilitation. Schizophr Res.

[36] Jones RB, Atkinson JM, Coia DA, Paterson L, Morton AR, McKenna K, Craig N, Morrison J, Gilmour WH (2001). Randomised trial of personalised computer based information for patients with schizophrenia. BMJ.

[37] Montes JM, Maurino J, Diez T, Saiz-Ruiz J (2010). Telephone-based nursing strategy to improve adherence to antipsychotic treatment in schizophrenia: A controlled trial. Int J Psychiatry Clin Pract.

[38] Frangou S, Sachpazidis I, Stassinakis A, Sakas G (2005). Telemonitoring of medication adherence in patients with schizophrenia. Telemed J E Health.

[39] Priebe S, McCabe R, Bullenkamp J, Hansson L, Lauber C, Martinez-Leal R, R&ouml;ssler W, Salize H, Svensson B, Torres-Gonzales F, van DBR, Wiersma D, Wright DJ (2007). Structured patient-clinician communication and 1-year outcome in community mental healthcare: cluster randomised controlled trial. Br J Psychiatry.

[40] Proudfoot J, Parker G, Manicavasagar V, Hadzi-Pavlovic D, Whitton A, Nicholas J, Smith M, Burckhardt R (2012). Effects of adjunctive peer support on perceptions of illness control and understanding in an online psychoeducation program for bipolar disorder: a randomised controlled trial. J Affect Disord.

[41] Todd NJ, Jones SH, Hart A, Lobban FA (2014). A web-based self-management intervention for Bipolar Disorder 'living with bipolar': a feasibility randomised controlled trial. J Affect Disord.

[42] Park KM, Ku J, Choi SH, Jang HJ, Park JY, Kim SI, Kim JA (2011). A virtual reality application in role-plays of social skills training for schizophrenia: a randomized, controlled trial. Psychiatry Res.

[43] Hansson L, Svensson B, Björkman T, Bullenkamp J, Lauber C, Martinez-Leal R, McCabe R, Rössler W, Salize H, Torres-Gonzales F, van DBR, Wiersma D, Priebe S (2008). What works for whom in a computer-mediated communication intervention in community psychiatry? Moderators of outcome in a cluster randomized trial. Acta Psychiatr Scand.

[44] Simon GE, Ludman EJ, Unützer J, Bauer MS, Operskalski B, Rutter C (2005). Randomized trial of a population-based care program for people with bipolar disorder. Psychol Med.

[45] Simon GE, Ludman EJ, Bauer MS, Unützer J, Operskalski B (2006). Long-term effectiveness and cost of a systematic care program for bipolar disorder. Arch Gen Psychiatry.

[46] Kuosmanen L, Välimäki Maritta, Joffe G, Pitkänen Anneli, Hätönen Heli, Patel A, Knapp M (2009). The effectiveness of technology-based patient education on self-reported deprivation of liberty among people with severe mental illness: a randomized controlled trial. Nord J Psychiatry.

[47] Wing JK, Beevor AS, Curtis RH, Park SB, Hadden S, Burns A (1998). Health of the Nation Outcome Scales (HoNOS). Research and development. Br J Psychiatry.

[48] Nuechterlein KH, Green MF, Kern RS, Baade LE, Barch DM, Cohen JD, Essock S, Fenton WS, Frese FA, Gold JM, Goldberg T, Heaton Robert K, Keefe Richard S E, Kraemer Helena, Mesholam-Gately Raquelle, Seidman Larry J, Stover Ellen, Weinberger Daniel R, Young Alexander S, Zalcman Steven, Marder Stephen R (2008). The MATRICS Consensus Cognitive Battery, part 1: test selection, reliability, and validity. Am J Psychiatry.

[49] Beebe LH, Smith K, Crye C, Addonizio C, Strunk DJ, Martin W, Poche J (2008). Telenursing intervention increases psychiatric medication adherence in schizophrenia outpatients. J Am Psychiatr Nurses Assoc.

[50] Španiel Filip, Hrdlička Jan, Novák Tomáš, Kožený Jiří, Höschl Cyril, Mohr P, Motlová Lucie Bankovská (2012). Effectiveness of the information technology-aided program of relapse prevention in schizophrenia (ITAREPS): a randomized, controlled, double-blind study. J Psychiatr Pract.

[51] Twamley EW, Jeste DV, Bellack AS (2003). A review of cognitive training in schizophrenia. Schizophr Bull.

[52] McGurk SR, Twamley EW, Sitzer DI, McHugo GJ, Mueser KT (2007). A meta-analysis of cognitive remediation in schizophrenia. Am J Psychiatry.

[53] Schultheis MT, Rizzo AA (2001). The application of virtual reality technology in rehabilitation. Rehabilitation Psychology.

[54] Välimäki Maritta, Hätönen Heli M, Lahti ME, Kurki M, Hottinen A, Metsäranta Kiki, Riihimäki Tanja, Adams CE (2014). Virtual reality for treatment compliance for people with serious mental illness. Cochrane Database Syst Rev.

[55] Grist R, Cavanagh K (2013). Computerised cognitive behavioural therapy for common mental health disorders, what works, for whom under what circumstances? A systematic review and meta-analysis. Journal of Contemporary Psychotherapy.

[56] Richards D, Richardson T (2012). Computer-based psychological treatments for depression: a systematic review and meta-analysis. Clin Psychol Rev.

[57] Rotondi AJ, Sinkule J, Haas GL, Spring MB, Litschge CM, Newhill CE, Ganguli R, Anderson CM (2007). Designing websites for persons with cognitive deficits: Design and usability of a psychoeducational intervention for persons with severe mental illness. Psychol Serv.

[58] Free C, Phillips G, Watson L, Galli L, Felix L, Edwards P, Patel V, Haines A (2013). The effectiveness of mobile-health technologies to improve health care service delivery processes: a systematic review and meta-analysis. PLoS Med.

[59] Naslund JA, Marsch LA, McHugo GJ, Bartels SJ (2015). Emerging mHealth and eHealth interventions for serious mental illness: a review of the literature. J Ment Health.

[60] Bakker D, Kazantzis N, Rickwood Debra, Rickard N (2016). Mental Health Smartphone Apps: Review and Evidence-Based Recommendations for Future Developments. JMIR Ment Health.

[61] Leach LS, Christensen H (2006). A systematic review of telephone-based interventions for mental disorders. J Telemed Telecare.

[62] Ben-Zeev D, Davis KE, Kaiser S, Krzsos I, Drake RE (2013). Mobile technologies among people with serious mental illness: opportunities for future services. Adm Policy Ment Health.

[63] Chaudhry SI, Mattera JA, Curtis JP, Spertus JA, Herrin J, Lin Z, Phillips CO, Hodshon BV, Cooper LS, Krumholz HM (2010). Telemonitoring in patients with heart failure. N Engl J Med.

[64] Jaana M, Paré Guy, Sicotte C (2009). Home telemonitoring for respiratory conditions: a systematic review. Am J Manag Care.

[65] El-Mallakh P, Findlay J (2015). Strategies to improve medication adherence in patients with schizophrenia: the role of support services. Neuropsychiatr Dis Treat.

[66] Anderson C, Henner T, Burkey J (2013). Tablet computers in support of rural and frontier clinical practice. Int J Med Inform.

[67] Pal K, Eastwood SV, Michie S, Farmer AJ, Barnard ML, Peacock R, Wood B, Inniss JD, Murray E (2013). Computer-based diabetes self-management interventions for adults with type 2 diabetes mellitus. Cochrane Database Syst Rev.

[68] Apolinário-Hagen Jennifer, Kemper J, Stürmer Carolina (2017). Public Acceptability of E-Mental Health Treatment Services for Psychological Problems: A Scoping Review. JMIR Ment Health.

