# Supplementary material for: Application and Effectiveness of Telehealth to Support Severe Mental Illness Management: Systematic Review
Source: JMIR Ment Health. 2018 Nov 21;5(4):e62. doi: 10.2196/mental.8816 (PMC6314801; doi:10.2196/mental.8816)
Supplement: Multimedia Appendix 1 [file mental_v5i4e62_app1.pdf]

Multimedia Appendix 1. Participant characteristics of the included studies.

First author, country of origin, year, design, length of follow-up	Population, setting, total number randomized	Participant demographics	Illness severity
Beebe, USA, 2008, RCT, 12 weeks	Adults with schizophrenia from a community mental health center, 29; Telephone intervention problem solving (TIPS) = 15, TAU = 14.	Male (n, %) Intervention = 7 (54), TAU = 8 (67); Age (not separated by group) 52 years (range 25 to 69); Caucasian (n, %) TIPS = 8 (62), TAU = 8; African American (n, %) TIPS = 5 (38), TAU = 6 (50); Oral atypicals (n, %) TIPS = 10 (77), TAU = 9 (75), Depot typicals (n, %) TIPS = 4 (31), TAU = 1 (8), Antidepressants (n, %) TIPS = 7 (54), TAU = 4 (33), Antianxiety agents (n, %) TIPS = 3 (23), TAU = 5 (42), Antiparkinsonians (n, %) TIPS= 5 (38), TAU = 2 (17), Hypnotics (n, %) TIPS = 2 (15), TAU = 3 (25)	Not reported
Bellucci, USA, 2002, RCT, 8 weeks	Adults with schizoaffective disorder or schizophrenia, 34; CACR = 17, control = 17	Characteristics not separated by group. Age (mean) 42; Male (n, %) 16 (47.1%); An average of 3.1 psychiatric medications	SANS summary score: mean (SD) CACR = 13.7 (3.6) Control = 13.1 (3.8)
Benedict, USA, 1994, RCT, follow-up not reported	Adults with chronic schizophrenia from an outpatient day treatment center, 38; CACR= 16, TAU = 17	Male (n) Experimental = 8, Control = 9; Age (mean, SD) Experimental = 38.1 (11.6), Control = 39.5 (11.1); White/black Experimental = 14/2, Control = 15/2; Days hospitalized (mean, SD) Experimental = 269.0 (211.8), Control = 173.8 (200.7); Chlorpromazine equivalents (mean, SD) Experimental = 279.7 (196.6), Control = 346.1 (544.1); Benzotropine equivalents (mean, SD) Experimental = 2.8 (2.2), Control = 3.1 (2.7)	SANS (mean, SD) Experimental = 11.7 (3.5), Control = 11.7 (3.9); SAPS (mean, SD) Experimental = 9.6 (2.9), Control = 8.4 (3.3)
Burda, USA, 1994, RCT, 8 weeks	Adult inpatients with schizophrenia or schizoaffective disorder in a medical center, 69; CACR= 40, control = 29	Age (mean, SD) Experimental = 50 (12.51), control = 41.97 (8.16); Diagnosis (not separated by group): undifferentiated type schizophrenia (n=24), paranoid type schizophrenia (n=23), schizoaffective (n=22);	Not reported
Cavallaro, Italy, 2009, RCT, 12 weeks	Adults with schizophrenia at two psychiatric care centers, 100; CACR = 50, control = 50	Age (mean, SD) Experimental = 33.2 (9.5), Control = 34.2 (6.8); Illness duration in years (mean, SD) Experimental = 8.28 (6.7), Control = 8.08 (5.1)	PANSS-positive SRT+CRT = 10.8 (3.8) SRT+PBO = 10.05 (3.6); PANSS-negative SRT+CRT = 13.7 (4.5) SRT+PBO = 14.6 (4.3)
Chan, Hong Kong, 2010, RCT, 10 weeks	Older adults with chronic schizophrenia from a residential care setting, 29; VR = 14, Control = 15	Male (n, %) VR = 10 (83.3), Control = 8 (53.3); Age (mean, SD) VR = 66.4 (6.2), Control = 65.87 (5.54)	Not reported

First author, country of origin, year, design, length of follow-up	Population, setting, total number randomized	Participant demographics	Illness severity
D'Amato, France, 2011, RCT, 12 weeks	Adults inpatients with schizophrenia, 77; CACR = 39, control = 38	Male (n) CRT = 29, Non-CRT = 29; Age (mean, SD) CRT = 33.4 (6.9), Non-CRT = 32.2 (6.0)	PANSS CRT = 73.3 (11.6) Non-CRT = 75.7 (13.0)
Dickinson, USA, 2010, RCT, 12 weeks	Adults with schizophrenia or schizoaffective disorder attending a Veterans Integrated Services Network, and community psychiatry clinics, 67; CACR = 35, control = 32	All participants were diagnosed with schizophrenia or schizoaffective disorder using the Structured Clinical Interview for DSM-IV (SCID), information from the participants' mental health care providers, and medical records; Male n (%) Remediation = 23 (65.7), Control = 21 (75.0); Age (mean, SD) Remediation = 46.9 (6.6), Control = 48.5 (8.8); African American n (%) Remediation = 23 (65.7), Control = 15 (53.6); Receiving second-generation antipsychotic (versus low-dose first-generation antipsychotic or other) n (%) Remediation = 26 (74.3), Control = 19 (67.9)	SANS score (mean, SD) Remediation = 23.60 (8.32), Control = 27.11 (11.98)
Frangou, UK, 2005, RCT, 8 weeks	Adults with schizophrenia attending outpatient clinics, 108; @HOME = 36, TAU = 36, Pill counting group = 36	Male (n) Routine care = 13, Pill counting = 7, @HOME = 5; Age (mean, SD) Routine care = 47.2 (9.8), Pill counting = 49.6 (11.6), @HOME = 45.5 (9.6); Hospitalizations in previous 12 months (mean, SD) Routine care = 4.3 (5.9), Pill counting = 3.6 (2.7), @HOME = 2.8 (2.5)	PANSS (mean, SD) Routine care = 46.6 (15.9) Pill counting = 43.4 (15.5) @HOME = 43 (14.9)
Hansson, Multicenter (Spain, The Netherlands, UK, Sweden, Germany & Switzerland), 2008, RCT, 12 months	Adults with schizophrenia on caseloads of participating key workers, 507; DIALOG = 271, TAU = 236	Female (%) Intervention = 32.5, TAU = 35.2; Age (mean, SD) Intervention = 42.5 (11.3), TAU = 41.8 (11.6); Duration of illness (yr) Intervention = 15.2 (9.9) TAU = 16.6 (10.5)	PANSS (mean, SD) Intervention = 62.2 (17.4) TAU = 64.8 (19.8)
Hermanutz, Germany, 1991, RCT, 4 weeks	Adults with schizophrenia on admission wards, 30; CACR = 10, control = 10, "0" group = 10	All participants were treated with major tranquilizers (average daily neuroleptic dose in chlorpromazine equivalents 150-750 mg); Age (mean, SD) Experimental = 31.5 (18-52), Control = 30 (22-60), "0" = 31.5 (22-54); Illness duration in years (range) Experimental = 6 weeks to 2.6 years, Control = 3 weeks to 10 years, "0" = 5 weeks to 6 years	Not reported
Hogarty, USA, 2004, RCT, 12 & 24 months	Adults with schizophrenia or schizoaffective disorder from a medical center, 121; CACR = 67, Enriched supportive therapy (EST) group = 54	Characteristics not separated by group. Diagnosis (% of total sample) Paranoid schizophrenia = 56, Schizoaffective disorder = 30, Other schizophrenia = 14	Not reported

First author, country of origin, year, design, length of follow-up	Population, setting, total number randomized	Participant demographics	Illness severity
Jones, UK, 2001, RCT, 12 weeks	Adults with schizophrenia in community mental health services, 112; Computer only = 56, Nurse + computer = 28, Nurse only = 28	Characteristics not separated by group; Aged between 18 and 65 years; Male (%) 67	Not reported
Keefe, USA, 2012, RCT, 12 weeks	Adults with schizophrenia at one of nine sites of the Schizophrenia Trials Network, 53; CACR = 27, control = 26	Characteristics not separated by group; Age, mean (SD): 37 (10.27); Male /Female: 39/14; White/ Black/ Other: 30/18/5	CGI (mean, SD) Control = 3.62 (0.75) Treatment = 3.41 (0.84); PANSS (mean, SD) Control = 56.69 (15.35) Treatment = 55.70 (13.75)
Kuosmanen, Finland, 2009, RCT, follow-up not reported	Adults with schizophrenia on nine acute wards in two psychiatric hospitals, 311; IT education group = 100, Conventional education group = 106, TAU = 105	Female (%) IT = 39, Conventional education = 39, Standard care = 45; Age (mean, SD) IT = 37.3 (12.4), Conventional education = 39.1 (12.6), Standard care = 37.9 (12.8)	PANSS total score (mean, SD) IT = 70.9 (18.2) conventional = 70.1 (20.6) standard care 73.2 (23.4)
Kurtz, USA, 2007, RCT, 12 months	Adults with schizophrenia or schizoaffective disorder attending The Institute of Living and a community center, 42; CACR = 23, control = 19	Male (%) CR = 60, CS = 74; Age (mean, SD) CR = 36.7 (12.2), CS = 32.9 (9.3); Duration of Illness in years (mean, SD) CR = 11.0 (10.4), CS = 9.8 (6.3); Treated with antipsychotic medicine (%) CR = 91, CS = 95	Not reported
Lee, Korea, 2013, RCT, 12 weeks	Adults with schizophrenia referred to a psychiatric rehabilitation unit, 60; CACR = 30, control = 30	Male (n) CT = 16/14 UR = 17/13; Age (mean, SD) CT = 43.53 (4.87) UR = 43.46 (3.53); Duration of illness in years (mean, SD) CT = 17.75 (4.14) UR = 17.53 (3.03); Medication in chlorpromazine mg daily equivalents (mean, SD) CT = 316.58 (182.15) UR = 317.08 (182.23)	PANSS (mean, SD) Positive symptoms CT = 15.21 (3.65) UR = UR 15.20 (3.65); Negative symptoms CT = 15.43 (4.24) UR = 15.69 (4.69); General psychopathology CT = 33.29 (6.89) UR = 33.22 (6.78)
Madoff, USA, 1996, RCT, 1 week, 4 weeks & 12 weeks after discharge	Adult acute psychiatric inpatients, 42; Computer = 34, control = 21	Male (n, %) Computer = 17 (50), Control = 8 (38); Age (mean, SD) Computer = 35.6 (9.5), Control = 34.6 (9.6); White ethnic group (n, %) Computer = 22 (65), Control = 13 (65)	Not reported

First author, country of origin, year, design, length of follow-up	Population, setting, total number randomized	Participant demographics	Illness severity
Mak, Poland, 2013, RCT, 8 weeks	Adults with paranoid schizophrenia, 81; CACR = 41, control = 40	Male (n) CACR = 19, control = 18; Age (mean, SD) CACR = 34 (11.07), control = 39 (12.99); Illness duration (mean, SD) CACR = 9 (7.21), control = 11 (8.56); Olanzapine CACR = 18 Control = 16; Ziprasidone CACR = 5 Control = 12; Perazine CACR = 7 control = 9; Clozapinum CACR = 3 Control = 0; Quetiapinum CACR = 1 Control = 0; Amisulpridum CACR = 1 Control = 0; Risperidone CACR = 2 Control = 3; Zuclopenthixol CACR = 3 Control = 0	PANSS Positive scale (mean, SD) CACR = 9.10 (2.25) control = 14.45 (7.10); PANSS Negative scale CACR = 13.68 (5.99) control = 20.98 (7.07); General psychopathology scale CACR = 24.59 (7.14) control = 33.73 (9.22)
Montes, Spain, 2010, RCT, 16 weeks	Adults with schizophrenia treated at community mental health centers, 928; Intervention = 456, Control = 472	Male n (%) Intervention = 276 (65.1) Control = 288 (65.5); Age (mean, SD) Intervention = 40.7 (11.8) Control = 39.5 (11.5); Duration of illness in years (mean, SD) Intervention = 13.5 (9.7) Control = 12.7 (9.4); Treatment with atypical antipsychotics n (%) Intervention = 406 (96.0) Control = 425 (96.4); Schizophrenia subtype n (%) Paranoid Intervention = 309 (72.9) Control = 319 (72.7); Undifferentiated Intervention = 52 (12.3) Control = 51 (11.6); Disorganized Intervention = 18 (4.3) Control = 24 (5.5); Residual Intervention = 45 (10.6) Control = 45 (10.3)	CGI-SCH SI score (mean, SD) Positive symptoms Intervention = 2.7 (1.3) control = 2.7 (1.3); Negative symptoms Intervention = 3.3 (1.3) control = 3.2 (1.3); Global symptoms Intervention = 3.3 (1.2) control = 3.2 (1.2)
Park, Korea, 2011, RCT, 5 weeks	Adults inpatients with schizophrenia, 91; Social skills training-virtual reality (SST-VR) = 46, Social skills training-traditional role play (SST-TR) = 45	Female n (%) SST-VR = 17 (51.5), SST-TR = 13 (41.9); Age (mean, SD) SST-VR = 28.1 (7.7), SST-TR = 31.2 (7.7); Duration of illness in years (mean, SD) SST-VR = 6.0 (5.7) SST-TR = 5.9 (6.3); Medications n (%) Atypical antipsychotics SST-VR = 30 (90.9) SST-TR = 29 (93.5); Typical antipsychotics SST-VR = 6 (18.2) SST-TR = 5 16.5; Mood stabilizers SST-VR = 1 (3.0) SST-TR = 1 (3.2); Antidepressants SST-VR = 1 (3.0) SST-TR = 3 (9.7); Benzodiazepines SST-VR = 9 (27.3) SST-TR = 7 (22.6)	PANSS (mean, SD) total SST-VR = 73.3 (12.6) SST-TR = 71.4 (12.7); Positive symptoms SST-VR = 18.1 (5.3) SST-TR = 17.0 (5.1); Negative symptoms SST-VR = 18.9 (4.8) SST-TR = 18.4 (4.7); General symptoms SST-VR = 36.3 (8.6) SST-TR = 36.0 (8.9)

First author, country of origin, year, design, length of follow-up	Population, setting, total number randomized	Participant demographics	Illness severity
Priebe, centers in Spain, The Netherlands, UK, Sweden, Germany & Switzerland, 2007, RCT, 12 months	Adults with schizophrenia or related psychotic disorder treated in community psychiatric services, 507; DIALOG= 271, TAU = 236	Age in years, mean (SD) TAU = 41.8 (11.6), DAILOG = 42.5 (11.3); Female, n (%) TAU = 83 (35.2), DIALOG = 88 (32.5); Diagnosis n (%) undifferentiated schizophrenia TAU = 89 (37.7) DIALOG = 91 (33.6), Paranoid schizophrenia TAU = 63 (26.7) DIALOG = 89 (32.8), Catatonic schizophrenia TAU = 4 (1.7) DIALOG = 1 (0.4), Hebephrenic schizophrenia TAU = 10 (4.2) DIALOG = 7 (2.6), Schizoaffective manic TAU = 7 (3.0) DIALOG = 19 (7.0), Schizoaffective depression (moderate) TAU = 9 (3.8) DIALOG = 9 (3.3); Schizoaffective depression (severe) TAU = 2 (0.8) DIALOG = 3 (1.1); Schizoaffective bipolar disorder 9 (3.8) 15 (5.5); Delusional disorder TAU = 2 (0.8) DIALOG = 1 (0.4); Other non-organic psychotic disorders TAU = 41 (17.4) DIALOG = 36 (13.3); Length of illness, years, mean (SD) TAU = 15.2 (9.9) DIALOG = 16.6 (10.5)	PANSS sub-scale scores: mean (SD) Positive TAU = 14.6 (5.7) Intervention = 15.0 (5.8); Negative TAU = 15.7 (6.0) Intervention = 17.2 (6.9); General TAU = 31.8 (9.1) Intervention = 32.6 (10.1)
Proudfoot, Australia, 2012, RCT, 12 weeks & 6 months	Adults with bipolar disorder at the Black Dog Institute Mood Disorders Clinic, 419; Bipolar Education Program (BEP) = 139, BEP plus online support (BEP+IS) = 134, control = 134	Male n (%) BEP = 46 (33.1%), BEP + IS = 36 (26.9%), Control = 41 (30.6%); Age n (%) 18–29 BEP = 43 (30.9%) BEP + IS = 38 (28.4%) Control = 36 (26.9%), 30–39 BEP = 49 (35.3%) BEP + IS = 47 (35.1%) Control = 54 (40.9%), 40–49 BEP = 28 (20.1%) BEP + IS = 35 (26.1%) Control = 30 (22.4%), 50–59 BEP = 15 (10.8%) BEP + IS = 13 (9.7%) Control = 10 (7.5%), 60+ BEP = 4 (2.9%) BEP + IS = 1 (0.7%) Control = 4 (3%); Currently taking medication n (%) BEP = 114 (82.0%) BEP + IS = 115 (85.8%) Control = 114 (85.1%)	Not reported
Rass, USA, 2012, RCT, 5 weeks, 10 weeks & 20 weeks	Adults inpatients with schizophrenia or schizoaffective disorder, 48; CACR = 17, Active Control = 17, TAU = 10	Male (n) CR = 10, Active control = 11, TAU = 9; Age (mean, SD) CR = 37.2 (12.5), Active control = 45.4 (9.0), TAU = 43.9 (8.9); Illness duration (mean, SD) CR = 17.9 (11.6), Active control = 22.6 (12.1), TAU = 19.9 (9.3); Schizoaffective Disorder (n) CR = 9 Active control = 11 TAU = 2	PANSS Positive (mean, SD) CR = 15.2 (5.6) Active control = 16.5 (6.2) TAU = 13.6 (3.8); PANSS Negative (mean, SD) CR = 12.4 (5.5) Active control = 13.2 (5.4) TAU = 15.0 (3.9); PANSS General (mean, SD) CR = 27.3 (7.9) Active control = 30.6 (8.2) TAU = 26.0 (3.8)
Sartory, Germany, 2004, RCT, 3 weeks	Adults inpatients with chronic schizophrenia, 42; CACR = 21, TAU = 21	Male (n) CR = 14, TAU = 14; Age (mean, SD) CR = 32.2 (8.5), TAU = 31.6 (8.9); Illness duration (mean years, SD) CR = 5.5 (4.8), TAU = 6.8 (5.5); Chlorpromazine equiv. (mg) CR = 550.92 (323.9) TAU = 584.8 (318.1)	No reported

First author, country of origin, year, design, length of follow-up	Population, setting, total number randomized	Participant demographics	Illness severity
Simon, USA, 2005, RCT, 12 months	Adults with bipolar spectrum disorder at four group-model behavioral health clinics, 441; Intervention = 212, TAU = 229	Female n (%) Intervention = 144 (68) Usual Care = 157 (69); Age (mean, SD) Intervention = 44.1 (13.4), Usual Care = 44.3 (12.9); Caucasian n (%) Intervention = 184 (87), Usual Care = 206 (90); Type 1 bipolar disorder n (%) Intervention = 165 (78) Usual Care = 171 (75)	Baseline mania PSR mean (SD) Intervention = 2.77 (1.72) Usual care = 2.78 (1.60); Current psychotic symptoms n (%) Intervention = 33 (16) Usual care = 30 (13)
Simon, USA, 2006, RCT, 24 months	Adults with bipolar spectrum disorder at four group-model behavioral health clinics, 441; Intervention = 212, TAU = 229	Female n (%) Intervention = 144 (68) Usual Care = 157 (69); Age (mean, SD) Intervention = 44.1 (13.4), Usual Care = 44.3 (12.9); Caucasian n (%) Intervention = 184 (87), Usual Care = 206 (90); Type 1 bipolar disorder n (%) Intervention = 165 (78) Usual Care = 171 (75)	Baseline mania PSR mean (SD) Intervention = 2.77 (1.72) Usual care = 2.78 (1.60); Current psychotic symptoms n (%) Intervention = 33 (16) Usual care = 30 (13)
Španiel, Czech Republic, 2012, RCT, 12 months	Adults with schizophrenia or schizoaffective disorder at 36 outpatient psychiatric centers, 158; Active group = 79, control = 79	Female (%) Active = 41.3 Control = 46.5; Age (mean, SD) Active = 32.4 (9.8) Control = 33.7 (10.4); Illness duration in months (mean, SD) Active = 101.4 (69.7) Control = 112.5 (91.2); Diagnosis schizophrenia/ schizoaffective n/n Active = 52/23 Control = 52/19	CGI-S score (mean, SD) Active = 2.3 (0.7) Control = 2.3 (0.7)
Todd, UK, 2014, RCT, 3 & 6 months	Adults with bipolar disorder in the community, 122; 'Living with Bipolar' Intervention (LWB) = 61, control = 61	Male n (%) LWB = 16 (26%) WLC =18 (30%); Age in years (mean, SD) LWB = 42 (10.35) WLC = 45 (11.97); White British LWB = 55 (90%) WLC = 54 (88%); Diagnosis: Bipolar I LWB = 47 (77%) WLC = 39 (64%), Bipolar II LWB = 11 (18%) WLC = 19 (31%), Rapid Cycling LWB = 3 (5%) WLC = 3 (5%)	Internal States Scale (ISS) scores (mean, SD): ISS Perceived Conflict LWB = 171.00 (102.92) WLC = 157.33 (102.43), ISS Wellbeing LWB = 100.44 (78.22) WLC = 106.86 (75.84), ISS Activation n LWB = 130.07 (123.00) WLC = 139.10 (127.33), ISS Depression LWB = 86.32 (61.57) WLC = 76.90 (52.35)
Tsang, Hong Kong, 2013, RCT, Not reported	Adult inpatients with schizophrenia attending a vocational rehabilitation program, 95; VR = 33, Therapist-administered training (TA) = 32, control = 30	Male (%) VR = 28 TA = 60 CG = 44; Age (mean, SD) VR = 39.60 (7.96) TA = 40.76 (9.19) CG = 41.56 (9.94); Illness duration in years (mean, SD) VR = 11.40 (7.08), TA = 16.0 (8.73), CG = 16.64 (9.40); Medication (%) Typical VR = 28% TA = 32% CG = 36%, Atypical VR = 72% TA = 68% CG = 64%	*Not reported*

First author, country of origin, year, design, length of follow-up	Population, setting, total number randomized	Participant demographics	Illness severity
Vita, Italy, 2011, RCT, 24 weeks	Adults with schizophrenia in 3 rehabilitative centers, 84; Integrated Psychological Therapy (IPT-cog) = 26, CACR = 30, Non-cognitive rehabilitation (REHAB) = 28	Female (n) IPT-cog = 5 CACR = 11 REHAB = 10; Age in years (mean, SD) IPT-cog = 37.15 (9.10), CACR = 36.87 (11.40), REHAB = 43.00 (7.76); Duration of illness in years (mean, SD) IPT-cog = 14.94 (9.76) CACR = 14.80 (9.78) REHAB = 17.93 (9.68); Chlorpromazine equivalents in mg (mean, SD) IPT-cog = 674.08 (417.97) CACR = 600.17 (362.59) REHAB = 714.00 (445.32)	CGI-S (mean, SD) IPT-cog = 5.0 (0.63) CACR = 4.67 (0.75) REHAB = 4.71 (0.93); PANSS (mean, SD) Positive IPT-cog = 19.0 (4.45) CACR = 18.97 (5.91) REHAB = 19.68 (6.67), Negative IPT-cog = 28.73 (6.65) CACR = 22.27 (7.95) REHAB = 21.18 (7.33)
