# Supplementary material for: Application and Effectiveness of Telehealth to Support Severe Mental Illness Management: Systematic Review
Source: JMIR Ment Health. 2018 Nov 21;5(4):e62. doi: 10.2196/mental.8816 (PMC6314801; doi:10.2196/mental.8816)
Supplement: Multimedia Appendix 2 [file mental_v5i4e62_app2.pdf]

Multimedia Appendix 2. Descriptions of the interventions included in the review.

First author, country of origin, year, design, length of follow-up	Technology	Description of intervention and comparator
Beebe, USA, 2008, RCT, 12 weeks	Telephones	Participants received all usual care plus weekly telephone calls from a nurse over a period of 3 months. TAU consisted of the usual medications, physician visits, follow-up appointments, and care available at the community center.
Bellucci, USA, 2002, RCT, 8 weeks	Computers	Participants received computer-based cognitive training from a psychology intern. Training sessions were administered in a private office in two half-hour sessions for 8 weeks. The waiting-list control group took part in standard therapeutic activities in the day treatment program and received computer training at the end of the study
Benedict, USA, 1994, RCT, follow-up not reported	Computers	Participants received a mean of 14.4 (SD 1.09) 50-minute sessions of guided practice with six computer-based attention tasks administered by trained undergraduate research assistants. Three to five training sessions were scheduled per week. TAU participants received the same multidisciplinary, day-treatment program as experimental participants.
Burda, USA, 1994, RCT, 8 weeks	Computers	Participants received 24 half-hour sessions (or 12 h) of CACR training over 8 weeks. The TAU group did not use computers and participated in regular therapeutic activities on the ward.
Cavallaro, Italy, 2009, RCT, 12 weeks	Computers	Participants received neurocognitive exercises for 3 half-days a week for approx. 15 months. Sets of exercises were individually created for each participant on the basis of the quality of baseline neuropsychological assessment. The SRT+CRT condition consisted of three 1-h sessions a week for a period of 12 weeks, giving a total of 36 h. The control condition consisted of 1 h a week of computer-aided non-domain-specific activity and 2 extra h a week of standard rehabilitation program (SRT).
Chan, Hong Kong, 2010, RCT, 10 weeks	Virtual reality	Participants received a series of simulated tasks within a VR environment through video contact twice a week. Ten sessions, each 15 min long, with increasing level of difficulty, was provided by an occupational therapist and consisted of two activities. Participants in the control group received TAU and the VR program 3 months later.
D'Amato, France, 2011, RCT, 12 weeks	Computers	The CACR group received, in addition to standard treatment, 14 individual 2 hour sessions of training in selecting, executing, and monitoring cognitive operations delivered over a 7-week period by a psychologist.
Dickinson, USA, 2010, RCT, 12 weeks	Computers	The CACR group received 36 sessions of cognitive remediation. Time in individual sessions included practice of cognitive exercises (roughly two-thirds of each session) alternated with trainer prompts, queries and feedback, and strategy review. Control group participants received the same amount of time on computers with the same trainers as the treatment group, but received gaming activities with low cognitive demand.
Frangou, UK, 2005, RCT, 8 weeks	Electronic medication dispenser	@HOME participants used a medication dispenser with a Medication Event Monitoring System (MEMSIV®). The bottle cap recorded when the bottle was opened and data were transmitted to the research and clinical teams via the @HOME platform. Alerts were issued if participants took less than 50% of their prescribed medication over 1 week. The Pill Counting group consisted of pharmacists counting the numbers of tablets returned at the hospital visit. TAU received standard care.
Hansson, Multicenter (Spain, The Netherlands, UK, Sweden, Germany & Switzerland), 2008, RCT, 12 months	Computers	Key workers in the intervention group discussed issues with their participants using a computer-mediated procedure every 2 months. TAU continued with standard treatment delivered by their key worker.
