# Supplementary material for: Application and Effectiveness of Telehealth to Support Severe Mental Illness Management: Systematic Review
Source: JMIR Ment Health. 2018 Nov 21;5(4):e62. doi: 10.2196/mental.8816 (PMC6314801; doi:10.2196/mental.8816)
Supplement: Multimedia Appendix 3 [file mental_v5i4e62_app3.pdf]

[illegible]	Random sequence [illegible]	Allocation [illegible]	Blinding of participants and [illegible]	Blinding of outcome [illegible]	Incomplete outcome [illegible]	Selective [illegible]	Other bias
	Adequate sequence [illegible]	*Adequate allocation [illegible]*	*Was knowledge of allocation adequately [illegible]*	*Was knowledge of allocation adequately [illegible]*	*Were incomplete outcome data [illegible]*	*Are reports free of selective [illegible]*	*Was the study free of other [illegible]*
Beebe	Yes	?	No	Yes	Yes	Yes	Yes
Bellucci	?	?	No	Yes	?	Yes	Yes
Benedict	?	?	No	?	?	?	Yes
Burda	?	?	No	?	No	Yes	Yes
Cavallaro	Yes	?	No	No	?	Yes	Yes
Chan	?	?	No	?	No	Yes	Yes
D'Amato	?	?	No	Yes	Yes	Yes	Yes
Dickinson	Yes	?	No	Yes	No	Yes	Yes
Frangou	Yes	?	No	?	?	Yes	Yes
Hansson	Yes	Yes	No	Yes	Yes	Yes	Yes
Hemanutz	?	?	No	?	?	Yes	Yes
Hogarty	?	?	No	Yes	Yes	Yes	Yes
Jones	?	?	No	?	No	Yes	Yes
Keefe	?	?	No	Yes	Yes	Yes	Yes
Kuosmanen	?	Yes	No	No	No	Yes	Yes
Kurtz	?	?	No	Yes	?	No	Yes
Lee	Yes	?	No	Yes	Yes	Yes	Yes
Madoff et al, 1996	?	?	No	?	No	Yes	No
Mak	?	?	No	?	Yes	Yes	Yes
Montes	Yes	Yes	No	No	Yes	Yes	Yes
Park	?	?	No	Yes	Yes	Yes	Yes
Priebe	Yes	Yes	No	Yes	Yes	Yes	Yes
Proudfoot	Yes	Yes	No	?	Yes	Yes	Yes
Ross	Yes	?	No	Yes	No	Yes	Yes
Sattory	?	?	No	Yes	Yes	Yes	Yes
Simon	Yes	Yes	No	Yes	Yes	Yes	Yes
Simon	Yes	Yes	No	Yes	No	Yes	Yes
Spaniel	?	Yes	Yes	Yes	Yes	No	Yes
Todd	Yes	Yes	No	Yes	Yes	Yes	Yes
Tsang	Yes	?	No	Yes	No	Yes	Yes
Yrita	Yes	Yes	No	Yes	Yes	Yes	Yes
