# Supplementary material for: Application and Effectiveness of Telehealth to Support Severe Mental Illness Management: Systematic Review
Source: JMIR Ment Health. 2018 Nov 21;5(4):e62. doi: 10.2196/mental.8816 (PMC6314801; doi:10.2196/mental.8816)
Supplement: Multimedia Appendix 4 [file mental_v5i4e62_app4.pdf]

Author	Technolog y	Intervention	Behavioural outcomes	Psychosocial outcomes	Cognitive outcomes	Conclusions
Beebe et al., 2008	Telephone	Weekly nurse support	Medication adherence: TIPS showed higher adherence to psychiatric medication after 12 weeks ($p < .05$)	*Not measured*	*Not measured*	Improved pill count adherence to psychiatric medication after telephone support
Bellucci et al., 2002	Computer	CACR	*Not measured*	No difference in self-esteem scores between the two groups. CACR showed greater reduction on SANS subscales: affective flattening ($p<0.05$); anhedonia ($p<0.01$); attentional impairment ($p<0.05$) and SANS summary score ($p<0.001$)	Higher cognitive functioning for logical memory thematic units ($p<0.01$), recall units ($p<0.05$) & trail making errors ($p<0.05$); no significant differences in verbal paired associates, digit span, MMSE, or other logical memory and trail making constructs	CACR training yielded significant improvement on several measures of cognitive functioning with similar effects on negative symptoms
Benedict et al., 1994	Computer	CACR	*Not measured*	*Not measured*	Reduced attentional capacity following attention training, compared to TAU ($p<0.05$)	Attention remediation did not lead to enhanced attentional capacity in schizophrenia

Author	Technology	Intervention	Behavioural outcomes	Psychosocial outcomes	Cognitive outcomes	Conclusions
Burda et al., 1994	Computer	CACR	*Not measured*	*Not measured*	Reduction in cognitive problems (*p*<0.01), improvement in memory scores (*p*<0.01) and improvement in trail making (p<0.01) after CACR, compared to TAU	Significantly fewer perceived cognitive problems after CACR training and improved in visuomotor sequencing skills.
Cavallaro et al., 2009	Computer	CACR	*Not measured*	Psychopathology remained unchanged between the two groups, QoL total score greater after CACR (*p*=0.03) and self directedness subscore (*p*=0.004)	CACR showed fewer number of CPT targets missed (*p*=0.03) and the number of perseverative errors on the WCST (*p*=0.04).	Domain-specific CACR training improved cognitive flexibility and sustained attention performances, compared to TAU.
Chan et al., 2010	Virtual reality (VR)	Cognitive training	*Not measured*	*Not measured*	Greater improvement in VR group for repetition (*p*<.001) and memory (*p*<.040)	An adapted VR program may enhance the cognitive functioning and volition of older adults with chronic schizophrenia

Author	Technology	Intervention	Behavioural outcomes	Psychosocial outcomes	Cognitive outcomes	Conclusions
D'Amato et al., 2011	Computer	CACR	*Not measured*	No significant improvements in quality of life, social autonomy, PANSS subscales, or CGI	Improved attention/ vigiliance (CPT-ID 2 digits *p*=0.004; 4 digits *p*=0.02) verbal working memory (*p*=0.04), verbal learning memory (WLM first recall *p*=0.002; percent of discrimination *p*=0.007) and reasoning and problem-solving (*p*=0.0004)	CACR improved attention/vigilance, verbal working memory, verbal learning memory and reasoning and problem solving. CACR had no effect on non-verbal working memory, learning, speed of processing, or psychosocial outcomes
Dickinson et al., 2010	Computer	CACR	*Not measured*	No significant effects of treatment on any symptom scale	No significant effects of treatment on neurocognitive functioning or perceived cognitive performance	CACR had no benefit for neuropsychological functioning, instrumental activities of daily living, or interpersonal effectiveness after 3 months

Author	Technology	Intervention	Behavioural outcomes	Psychosocial outcomes	Cognitive outcomes	Conclusions
Frangou et al., 2005	Remote sensor on pill bottle	Remote medication monitoring (@HOME)	Medication adherence: Adherence was significantly better for @HOME than in either control (*p*=0.001) or pill counting (*p*=0.007); Healthcare utilisation: fewer medical and emergency visits in the @HOME group at study end-point (*p*=0.002)	Control group showed significantly less improvement in PANSS scores than Pill Counting (*p*=0.008) and @HOME (*p*=0.04). CGI ratings were lower in @HOME compared to control group (*p*=0.01) and pill counting (*p*=0.04), with pill counting showing the most improvement (*p*=0.05).	*Not measured*	The use of @HOME in adults suffering from schizophrenia was found to have high participant and clinician acceptability and translated into palpable benefits in clinical outcomes and service use.
Hansson et al., 2008	Hand held computers	Technology supported consultations with key workers (DIALOG)	*Not measured*	Better relationship with the key worker (*p*=0.004) and shorter duration of illness (*p*=0.04) for DIALOG. Participants with competitive employment (*p*=.047) and shorter illness duration (*p*=0.002) showed greater reduction of unmet needs; Older	*Not measured*	Participants with a shorter duration of illness and a better baseline perceived helping alliance receiving DIALOG improved more in quality of life than TAU. Participants with a competitive employment and a shorter duration of illness benefited more from the

Author	Technology	Intervention	Behavioural outcomes	Psychosocial outcomes	Cognitive outcomes	Conclusions
				participants had better treatment satisfaction (*p*=0.004) after DIALOG. Participants with more severe negative symptoms at baseline improved more regarding quality of life (*p*=0.022). A main effect on reduction of unmet needs for participants with a more severe initial psychopathology (*p*=0.001)		DIALOG intervention than TAU. Older participants in the DIALOG intervention group had better treatment satisfaction.
Hermanutz et al., 1991	Computer	CACR	*Not measured*	No group effects on mood & paranoid tendencies, or social professional & family functioning	A significant group effect on attention-stress test (*p*<0.05), but no other cognitive measures.	Improvement in information processing cannot always be made visible through clinical and psychometric methods

Author	Technology	Intervention	Behavioural outcomes	Psychosocial outcomes	Cognitive outcomes	Conclusions
Hogarty et al., 2004	Computer	CACR	*Not measured*	At 12 months, CACR showed significant improvement in social adjustment (*p*=0.046). At 24 months significant differential improvement for CACR was seen in social cognition (*p*=0.001) & social adjustment (*p*=0.01)	At 12 months, CACR showed a benefit for processing speed (*p*<0.001) and neurocognition (*p*=0.003). At 24 months, CACR improved processing npeed (p<0.001), neurocognition (p=0.02) & cognitive style (p=0.001)	CACR demonstrated significant differential effects on all domains of behavior and cognition except residual symptoms. Modest CACR effects on the behavioral composites at 1 year
Jones et al., 2001	Computer	Patient education	*Not measured*	No significant differences between the 3 groups in mental state, or insight	No significant differences between the 3 groups in schizophrenia knowledge or neurocognitive functioning	Personalised, computer based health education for adults with schizophrenia was acceptable and as effective as educational sessions given by a nurse. However, the computer-based intervention had no advantage for costs.

Author	Technology	Intervention	Behavioural outcomes	Psychosocial outcomes	Cognitive outcomes	Conclusions
Keefe et al., 2012	Computer	CACR	*Not measured*	No significant differences between the 2 groups in mental state, self-esteem or social cognition, or schizophrenia symptoms.	CACR significantly improved auditory discrimination after 20 sessions (*p*=0.0005) and at the end of treatment (*p*=0.0009); MCCB composite score significantly improved after 20 sessions OF CACR (*p*=0.0470), with verbal learning contributing most strongly to this improvement (*p*<.05)	CACR demonstrated significant improvement in cognitive outcomes after 20 sessions of treatment
Kuosmanen et al., 2009	Computer	Patient education	*Not measured*	No significant differences between the groups in perceived deprivation of liberty or satisfaction	*Not measured*	IT education was not superior compared with other means of patient education methods for reducing perceived deprivation of liberty

Author	Technology	Intervention	Behavioural outcomes	Psychosocial outcomes	Cognitive outcomes	Conclusions
Kurtz et al., 2007	Computer	CACR	*Not measured*	*Not measured*	An advantage for CACR in working memory (*p*=.027), verbal episodic memory (*p*<0.001), spatial episodic memory (*p*=0.002), processing speed (*p*<0.001) and reasoning/executive function (*p*<0.001)	CACR provides incremental benefit for neurocognition, but that exposure to a computer, interaction with a clinician and non-specific cognitive challenge produce non-specific improvement in neurocognitive
Lee et al., 2013	ComputerCACR	CACR	CACR had a better effect on work quality (*p*<0.01) and work habits (*p*<0.05).	No significant differences between the two groups on PANSS scores	CACR showed significant improvements in continuous performance "hit rate" (*p*<0.001), continuous performance sensitivity (*p*<0.001), digit span forward (*p*<0.001) and digit span backward (*p*<0.05)	CACR showed a beneficial effect on CPT, digit span and work behavior but not psychopathology

Author	Technology	Intervention	Behavioural outcomes	Psychosocial outcomes	Cognitive outcomes	Conclusions
Madoff et al., 1996	Computer	Patient education	Medication adherence: No significant differences between the two groups in adherence to medication at 1 week, 4 weeks or 12 weeks.	No significant decrease in relapse rates	No significant difference in increase in medication knowledge	The computer group performed at least as well as the control group on all measures.
Mak et al., 2013	ComputerCACR	CACR	*Not measured*	*Not measured*	No significant changes in scores in TMT, Stroop and WCST parameters as a result of CACR	No significant differences in neuropsychological outcomes between the groups
Montes et al., 2010	Telephone	Nurse assessment and psychiatrist appointments	Medication adherence Significantly more adults in the telephone group were adherent to treatment compared to TAU after 16 weeks (*p*=0.0007).	Significantly better DAI-10 scores (*p*<0.0001) and significantly lower mean score in the positive symptoms subscale of CGI-SCH-SI than TAU (*p*=0.02). No differences in the other four subscales of the CGISCH-SI or quality of life	*Not measured*	Among stable outpatients with schizophrenia, a telephone-based strategy provided a significant advantage improving medication adherence over usual care

Author	Technology	Intervention	Behavioural outcomes	Psychosocial outcomes	Cognitive outcomes	Conclusions
Park et al., 2011	Virtual reality (VR)	Social skills training	SST-VR group had greater improvement in conversational skills than SST-TR group (*p*<0.001), but SST-TR showed greater improvement in the nonverbal skills (*p*=0.015). SST-VR group showed greater improvement in assertiveness (*p*=0.030).	No significant differences were found between the groups in social problem-solving or relationship change	*Not measured*	The SST interventions produced meaningful improvement regardless of the type of SST. The use of VR produced greater improvement in conversational skills, but not for vocal and nonverbal skills.
Priebe et al., 2007	Hand held computers	Technology supported consultations with key workers (DIALOG)	*Not measured*	DIALOG showed significantly higher quality of life (*p*=0.04), fewer unmet needs (*p*=0.04) and higher treatment satisfaction (*p*=0.01) compared with TAU. No significant differences in positive or negative symptoms between the groups	*Not measured*	After 12 months, the DIALOG intervention had a significant positive effect on quality of life, unmet needs for care and treatment satisfaction

Author	Technology	Intervention	Behavioural outcomes	Psychosocial outcomes	Cognitive outcomes	Conclusions
Proudfoot et al., 2012	Website	Online bipolar education program + online peer support (BEP+IS) versus online bipolar education program + attentional control (BEP)	*Not measured*	No significant differences in illness perceptions, mood, work and social adjustment, self esteem, satisfaction with life, multidimensional health locus of control or stigma between the two groups	*Not measured*	Significant improvements in from pre- to post-intervention, were found across all groups.
Rass et al., 2012	Computer	CACR	*Not measured*	*Not measured*	No significant differences in letter fluency, marginal interaction HVLT recognition discrimination, or category fluency. Significantly greater BVMT total, delay, marginally greater letter fluency, global cognition, MASQ visual perceptual ability, and MASQ verbal memory in the	Participants in all groups improved in information processing, verbal memory and visuospatial memory. No significant differences were found when controlling for IQ (covariate).

Author	Technology	Intervention	Behavioural outcomes	Psychosocial outcomes	Cognitive outcomes	Conclusions
					TAU group compared to CACR.	
Sartory et al., 2004	Computer	CACR	*Not measured*	*Not measured*	The CACR group showed greater improvement in immediate recall ($p<0.05$), delayed recall ($p<0.01$), digit symbol test ($p<0.05$) and word fluency ($p<0.05$)	Computerised cognitive remediation leads to significant improvements in verbal memory, processing speed & executive function in adults with schizophrenia

Author	Technolog y	Intervention	Behavioural outcomes	Psychosocial outcomes	Cognitive outcomes	Conclusions
Simon et al., 2005	Telephone	Medication monitoring	Healthcare utilisation: Telephone group made significantly more psychiatric visits for medication management (*p*=0.01) and were more likely to use one or more atypical antipsychotics for at least 90 days (*p*=0.05) The two groups did not differ in use of mood stabilizer or antidepressant drugs.	No significant differences in reduction of mania scores over 12 months (*p*=0.70). Depressive symptoms in the intervention group showed a larger decline over time than those in the usual care group (*p*=0.043)	*Not measured*	The intervention led to significant reductions in severity of mania symptoms and time spent in manic episodes over 12 months
Simon et al., 2006	Telephone	Medication monitoring	*Not measured*	Mania scores were significantly lower after telephone monitoring (*p*=.04). No significant differences between the groups in depression	*Not measured*	The systematic care programme reduced the frequency and severity of manic symptoms.

Author	Technology	Intervention	Behavioural outcomes	Psychosocial outcomes	Cognitive outcomes	Conclusions
Spaniel et al., 2012	Remote monitoring	SMS-prompted relapse prevention (ITAREPS)	Healthcare utilisation: Significant fewer inpatient days for the intervention group (p<0.05)	*Not measured*	*Not measured*	Relapse prevention programmes based on SMS technologies may represent a novel, cost-effective and simple method for reducing disease burden and inpatient costs
Todd et al., 2014	Website	Online self-management intervention for bipolar disorder	Healthcare utilisation: Medication and service use reduced on average during the trial in LWB group and increased on average for the WLC group, statistical analysis not reported.	Physical quality of life (p=0.01) and psychological quality of life (p=0.02), recovery (p<0.01), perceived conflict (p=0.03) and social function (p<0.01) all improved significantly more in LWB than in WLC	*Not measured*	This trial has provided preliminary evidence that a web-based treatment approach in Bipolar Disorder is feasible and potentially effective
Tsang et al., 2013	Virtual reality (VR)	Vocational training	Work performance: Both the VRG (p=0.03) and the TAG (p<0.01) showed significantly better improvements in on-site work performance than	No significant differences between the groups in self-efficacy	VR group showed greater improvement in attention (p=0.03), memory (p=0.02) and executive functioning-percentage of error (p<0.001)	Participants who received VR showed improvements in cognitive functioning, self-efficacy and work performance

Author	Technology	Intervention	Behavioural outcomes	Psychosocial outcomes	Cognitive outcomes	Conclusions
			controls		and percentage of conceptual level response (p<0.001)	
Vita et al., 2011	Computer	CACR	*Not measured*	CACR had significantly better HONOS outcome than the REHAB and the IPT-cog groups (p<0.001). Both groups treated with cognitive remediation had greater improvement in PANSS positive, negative and total scores, but not CGI-S scores than the REHAB group	Compared with TAU, the IPT-cog group had greater improvement in processing speed (p=0.028) and working memory (p=0.020) after 24 weeks. No significant differences between the groups in neurocognitive measures	Participants undergoing cognitive remediation demonstrated significantly greater improvements in psychopathologic, cognitive and functional outcomes

TM = Trail making test from the Halstead – Reitan Neurological Test Battery, WMS-III = Wechsler memory scale-III, MMSE = Mini-mental status exam, SANS = Scale for the assessment of negative symptoms, SES = Self-esteem scale, CGI-SCH = Clinical Global Impression- Schizophrenia, CGI-SCH-SI = Clinical Global Impression- Schizophrenia severity of illness subscale
